# Remotely Delivered Exercise-Based Cardiac Rehabilitation: Design and Content Development of a Novel mHealth Platform

**DOI:** 10.2196/mhealth.5501

**Published:** 2016-06-24

**Authors:** Jonathan C Rawstorn, Nicholas Gant, Andrew Meads, Ian Warren, Ralph Maddison

**Affiliations:** ^1^ National Institute for Health Innovation University of Auckland Auckland New Zealand; ^2^ Department of Exercise Sciences University of Auckland Auckland New Zealand; ^3^ Department of Computer Science University of Auckland Auckland New Zealand

**Keywords:** telemedicine, telerehabilitation, wireless technology, remote sensing technology, behavioral medicine, myocardial ischemia

## Abstract

**Background:**

Participation in traditional center-based cardiac rehabilitation exercise programs (exCR) is limited by accessibility barriers. Mobile health (mHealth) technologies can overcome these barriers while preserving critical attributes of center-based exCR monitoring and coaching, but these opportunities have not yet been capitalized on.

**Objective:**

We aimed to design and develop an evidence- and theory-based mHealth platform for remote delivery of exCR to any geographical location.

**Methods:**

An iterative process was used to design and develop an evidence- and theory-based mHealth platform (REMOTE-CR) that provides real-time remote exercise monitoring and coaching, behavior change education, and social support.

**Results:**

The REMOTE-CR platform comprises a commercially available smartphone and wearable sensor, custom smartphone and Web-based applications (apps), and a custom middleware. The platform allows exCR specialists to monitor patients’ exercise and provide individualized coaching in real-time, from almost any location, and provide behavior change education and social support. Intervention content incorporates Social Cognitive Theory, Self-determination Theory, and a taxonomy of behavior change techniques. Exercise components are based on guidelines for clinical exercise prescription.

**Conclusions:**

The REMOTE-CR platform extends the capabilities of previous telehealth exCR platforms and narrows the gap between existing center- and home-based exCR services. REMOTE-CR can complement center-based exCR by providing an alternative option for patients whose needs are not being met. Remotely monitored exCR may be more cost-effective than establishing additional center-based programs. The effectiveness and acceptability of REMOTE-CR are now being evaluated in a noninferiority randomized controlled trial.

## Introduction

Coronary heart disease (CHD) is a leading cause of mortality and morbidity worldwide. CHD accounts for 1 in 7 deaths in the United States, approximately 1 in 20 adults have diagnosed CHD, and its prevalence is expected to increase ≈18% by 2030 [1]. Furthermore, people with CHD account for more than 40% of all CHD events, more than 35% of nonfatal myocardial infarction, and more than 50% of fatal coronary events [2]. This emphasizes the need for effective secondary prevention programs that target modifiable cardiovascular risk factors in order to reduce the risk of recurrent cardiac events and mortality.

Cardiac rehabilitation (CR) is an essential component of contemporary cardiac care, and exercise training is consistently identified as a central element in international guidelines [3-5]. Exercise-based cardiac rehabilitation (exCR) is commonly delivered in hospitals and rehabilitation clinics (center-based exCR) and should include an individualized, progressive program of aerobic and strength training that accounts for patients’ clinical status, risk stratification, comorbidities, and goals [4]. ExCR improves all-cause and cardiac mortality, recurrent cardiac event risk, several modifiable cardiovascular risk factors, and exercise capacity [5-10], and is more cost-effective for reducing premature death than most common pharmacological and surgical interventions [11]. However, referral rates and uptake of exCR are low [11-13], and participation is commonly limited by program availability, transport restrictions, inconvenient program scheduling, and domestic or occupational responsibilities [14,15]. These barriers suggest accessibility is a primary factor limiting utilization of traditional center-based exCR programs, and overcoming participation barriers should be prioritized, as nonattendance is associated with poorer risk factor knowledge and risk profile [16].

Home-based exCR programs enhance accessibility [17] and provide comparable health benefits [18], but cannot deliver the supervision, feedback, and individualized coaching provided by exCR specialists during center-based exCR. These limitations preclude optimal individualization of exercise prescription and may limit improvements in modifiable cardiovascular risk factors and functional capacity [19,20].

Information and communication technologies offer opportunities to augment home-based exCR by connecting exCR specialists with patients outside the clinical environment. Telehealth-assisted programs have commonly used fixed-line telephones, email, and mobile phone short message service (SMS) to deliver motivational support, behavioral change counseling, and feedback about goal achievement or exercise adherence [21-31]. Additional telehealth interaction between patients and exCR specialists augments early home-based exCR programs, and secondary prevention effects appear promising. However, constraints of commonly used telehealth technologies have limited program flexibility and are unable to emulate the exercise supervision, coaching, and individualization, which are typical of center-based exCR. Some telehealth exCR platforms have enabled patients to upload accelerometer or heart rate data to Web-based portals to be reviewed by exCR specialists [22,24-26,30,31]. However, asynchronous uploading of data has limited the utility of these platforms to high-level review of physical activity and adherence.

Telehealth exCR has, to date, been unable to provide exercise monitoring, feedback, coaching, and individualized exercise prescription in a manner consistent with best practice international CR guidelines; however, rapid advances in mobile sensor and communication technologies can help bridge this gap. Increasingly powerful smartphones, rapid mobile broadband connection speeds, and interoperable wearable sensors mean the technological capability for real-time remote exercise monitoring and coaching is now readily available. Smartphones are appealing for health intervention delivery as they enable individualized and timely provision of intervention content [12]. Moreover, rapid growth in smartphone mobile phone ownership (≈75% in many countries), mobile broadband subscriptions (80% in developed countries), and coverage (70% and 90% of the total and urban global population, respectively) [32-34] suggest the time is right to introduce more sophisticated telehealth exCR platforms that can optimize program flexibility, accessibility, responsiveness, and individualization. This is supported by results from several studies that indicate telehealth exCR has beneficial effects on modifiable cardiovascular risk factors [21-31], but intervention characteristics are likely constrained by telehealth platforms that do not enable sufficient individualization of exercise prescription or monitoring of exercise performance [27]. Furthermore, predominant reliance on fixed-line telephone communications in previous telehealth exCR interventions constrains patients within the home environment and limits program flexibility.

The feasibility of more advanced mobile health (mHealth) exCR platforms comprising wearable sensors, smartphones and real-time remote data transmission has been demonstrated [15,35], but only one evaluation study has been published.

Real-time remotely monitored exercise improves submaximal aerobic exercise capacity and health-related quality of life (HRQoL) for patients undergoing percutaneous coronary intervention; [26] however, real-time monitoring was only sustained for 2 weeks and it was unclear whether exCR specialists used the data to inform real-time individualized feedback or coaching. One of these platforms (REMOTE-CR) is currently being evaluated in a noninferiority randomized controlled trial comparing mHealth and center-based exCR [36].

Embracing advances in wearable sensor and mobile communication technologies can enable mHealth exCR to combine the universal accessibility of home-based exCR with the clinical expertise, supervision, and coaching that has traditionally been limited to center-based exCR. These opportunities have yet to be capitalized on and addressing them could substantially increase the reach of exCR by providing more flexible, responsive, and interactive alternatives to existing center- and home-based programs. This article describes the development of an mHealth exCR platform designed to address these limitations. The paper first defines the main platform design objectives and then describes how components of an mHealth CR development framework were integrated into the design process. Subsequent sections outline the platform design, including the technology components and intervention content development.

## Methods

### Design Objectives

The primary purpose of the mHealth exCR platform was to overcome accessibility barriers that limit center-based exCR participation while retaining the clinical expertise provided by exCR specialists during center-based programs. The major platform design objectives were to provide universal access to real-time exercise monitoring, coaching, and feedback and theory-based behavior change strategies. To achieve these objectives, we aimed to develop an alternative for the large number of CHD patients whose needs are not currently being fulfilled by center-based exCR programs.

We developed an mHealth exCR platform, named REMOTE-CR, that integrates smartphones, wearable sensors, and custom smartphone and Web-based apps to provide real-time remote exercise monitoring, evidence-based exercise prescription and coaching, theory-based behavior change education, and social support to CHD outpatients in almost any location. REMOTE-CR was designed to optimize ease of use and allow rapid scalability while adhering to evidence-based guidelines, as these characteristics will likely facilitate transition into practice [37].

### Development Framework

A recently proposed mHealth CR development and evaluation framework suggests CR interventions should address the core components of CR, apply behavior change theory, enable individual tailoring of features, demonstrate high usability, and be evaluated in a randomized controlled trial that assesses patient-centered outcomes [38]. The design, development, and subsequent evaluation of REMOTE-CR were based on this framework, with appropriate adaptations to suit the intended design objectives.

#### Core Components of Cardiac Rehabilitation

The core components of CR include patient evaluation, medical and lifestyle risk factor management, cardioprotective therapies, psychosocial management, exercise training, and health behavior change education [4,39]. Many traditional center-based exCR programs focus on exercise training and physical activity counseling, while other core components may be delivered via alternative channels such as outpatient clinics and group seminars. As REMOTE-CR is intended to provide an alternative to traditional center-based exCR programs, the platform features also focus on exercise training and physical activity behavior change. We aligned REMOTE-CR with the format of center-based exCR to increase the likelihood of transition into practice, an approach that has previously been recommended for the development of health behavior change interventions [37]. Although REMOTE-CR does not currently include additional core CR components, the platform architecture was designed to enable modular expansion in future iterations. The initial focus on exercise training and physical activity behavior change was intended to facilitate robust evaluation of remotely delivered exCR in comparison with center-based programs.

#### Behavior Change Theory

Theory-based interventions are considered more effective than those without theoretical underpinning, and integrating principles from behavior change theories into mHealth intervention design may significantly increase the likelihood of success [40,41]. Development of the REMOTE-CR platform and intervention content were informed by behavior change theories including Social Cognitive Theory and its key component self-efficacy [42], Self-determination Theory [43,44], and a taxonomy of behavior change techniques [45].

Self-efficacy refers to individuals’ perceptions that they can control their health behaviors [46]; higher levels of self-efficacy are expected to facilitate behaviors that help to maintain self-regulated motivation such as setting goals, creating incentives, and seeking social support [47]. Sources of self-efficacy information include performance accomplishment, vicarious experience, verbal persuasion, and physiological cues. Self-efficacy influences exercise initiation and maintenance [48,49], physical activity level [50], and clinical outcomes [51], is one of the most commonly examined psychological variables in the cardiac setting, and is a recommended intervention component in New Zealand CR guidelines [20].

Self-determination Theory proposes motivational orientation lies on a continuum anchored by intrinsic and extrinsic motivation, where an individual’s orientation is determined by effects of environmental and individual factors on the satisfaction of three basic psychological needs: autonomy, competence, and relatedness [43,44,52]. Briefly, intrinsic motivation refers to the pursuit of internal outcomes such as accomplishment, development, and satisfaction; conversely, extrinsic motivation refers to the pursuit of external outcomes such as tangible or social rewards [52]. Autonomy refers to a sense of regulating one’s own behavior, competence refers to a sense one’s actions, and relatedness refers to a sense of connection with others in a specific setting [43,44]. Competence shares fundamental similarities with self-efficacy as both constructs reference perceived confidence in task-specific success and are informed by performance accomplishment. Perceptions of autonomy, competence, and relatedness are influenced by physical and social environmental factors, individuals’ motives for undertaking exercise, and their propensity to pursue intrinsic or extrinsic directives [52]. A comprehensive meta-analysis examining the influence of self-determination on exercise behavior associated more self-determined motivational orientations with superior exercise adoption and long-term adherence [52]. Furthermore, some evidence suggests self-determined motivation has a greater effect on exercise adherence than self-efficacy [49].

While Social Cognitive Theory and Self-determination Theory serve as the theoretical constructs underlying design and development of REMOTE-CR, they do not provide tangible or identifiable intervention components, per se. A taxonomy of behavior change techniques has been developed to identify, define, and categorize active intervention components that are designed to alter processes underlying behavior regulation [45]. The taxonomy defines 93 behavior change techniques across 16 categories. Behavior change techniques that support REMOTE-CR’s underlying theoretical constructs (ie, self-efficacy and self-determination) were adapted as required to suit the technological requirements of the REMOTE-CR platform. In total, REMOTE-CR includes 24 behavior change techniques from 10 categories (Table 1). Behavior change techniques were integrated throughout the REMOTE-CR platform, including the intervention content and features of the patient-facing smartphone app and exCR specialist-facing Web-based app.

**Table 1:** REMOTE-CR behavior change techniques^a^.

	Category		Behavior change technique
1	Goals and planning				
		1.1^b,c^	Goal setting (behavior)
		1.2^d^	Problem solving
		1.3^a^	Goal setting (outcome)
		1.4^b,d^	Action planning
		1.5^b,c,d^	Review behavior goal(s)
		1.6^b,c^	Discrepancy between current behavior and goal
2	Feedback and monitoring				
		2.1^b,c^	Monitoring of behavior by others without feedback
		2.2^b,c^	Feedback on behavior
		2.3^b^	Self-monitoring of behavior
		2.4^b,d^	Self-monitoring of outcome(s) of behavior
		2.6^b^	Biofeedback
3	Social support					
		3.1^b,c,d^	Social support (unspecified)
		3.3^b,c,d^	Social support (emotional)
4	Shaping knowledge					
		4.1^c,d^	Instruction on a behavioral act
5	Natural consequences					
		5.1^c,d^	Information about health consequences
8	Repetition and substitution				
		8.1^b^	Behavioral practice/rehearsal
		8.6^d^	Generalization of a target behavior
		8.7^c^	Graded tasks
9	Comparison of outcomes				
		9.1^c,d^	Credible source
10	Reward and threat				
		10.4^c^	Social reward
		10.9^d^	Self-reward
13	Identity		
		13.2^d^	Framing/reframing
15	Self-belief				
		15.1^c^	Verbal persuasion about capability
		15.3^d^	Focus on past success

^a^Behavior change techniques defined by Michie et al [45].

^b^Patient-facing mobile phone app.

^c^exCR specialist-facing Web-based app.

^d^Behavior change education content.

#### Individualization and Usability

A review of mHealth behavior change suggests individualized interventions are more effective at changing behavior, although few interventions have implemented tailored components [53]. The accessibility of mobile platforms may encourage greater use of tailored features by patients [38], and the REMOTE-CR was designed to include several individualized components including exercise prescription, exercise performance feedback, exercise coaching, goal setting and achievement feedback, and social support. Further details about platform features are discussed in the following section.

CHD patients are mostly of older age; therefore, use of smartphones and wearable sensors presents substantial usability challenges that may affect adoption and adherence. Factors that facilitate mHealth exCR usability are not well defined [38]; however, features such as automated and/or wireless data entry promote ease of use [54]. REMOTE-CR features were designed to minimize the required user input in order to aid usability. The smartphone app was designed with a simple, intuitive user interface and navigational complexity was minimized. Most functions are automated, including tasks such as connecting the smartphone and wearable sensor, recording exercise data, and presenting goal achievement feedback. Furthermore, a custom middleware platform was implemented to manage low-level functions such as authentication, encryption, and connectivity. Appropriate training is also likely to alleviate usability challenges [55]. A face-to-face training module was developed to explain REMOTE-CR platform functionality. The module includes verbal instruction, demonstration, and hands on practice. The training module is supported by an illustrated user guide that comprehensively documents platform functionality, and ongoing email or telephone support when required.

#### Evaluation of Patient-Centered Outcomes

As REMOTE-CR has been designed to provide an alternative to existing center-based exCR it will be necessary to determine how the program compares with traditional programs before it can be recommended to patients. Therefore, a noninferiority randomized controlled trial comparing mHealth and center-based exCR was planned. This intended evaluation informed aspects of platform and intervention content design, such as exercise prescription, monitoring and coaching, intervention content, and intervention duration. The planned evaluation is in progress, and in-line with the adopted mHealth development framework [38], it will assess patient-centered outcomes such as participation, physical activity level, exercise capacity, modifiable cardiovascular risk factors, quality of life, cost, and cardiovascular events. Further details are provided in the trial protocol [36].

## Results

Previous mHealth exCR platforms have provided limited exercise monitoring, feedback, and coaching capabilities, and this may limit intervention effectiveness in terms of exercise-induced risk factor modification [27]. To address these limitations, REMOTE-CR integrates advances in wearable sensor and mobile communications technologies to provide enhanced exercise training and physical activity behavior change capabilities, similar to those provided by traditional center-based exCR programs. The REMOTE-CR platform design is described in detail below.

### Platform Components

**Figure 1:**
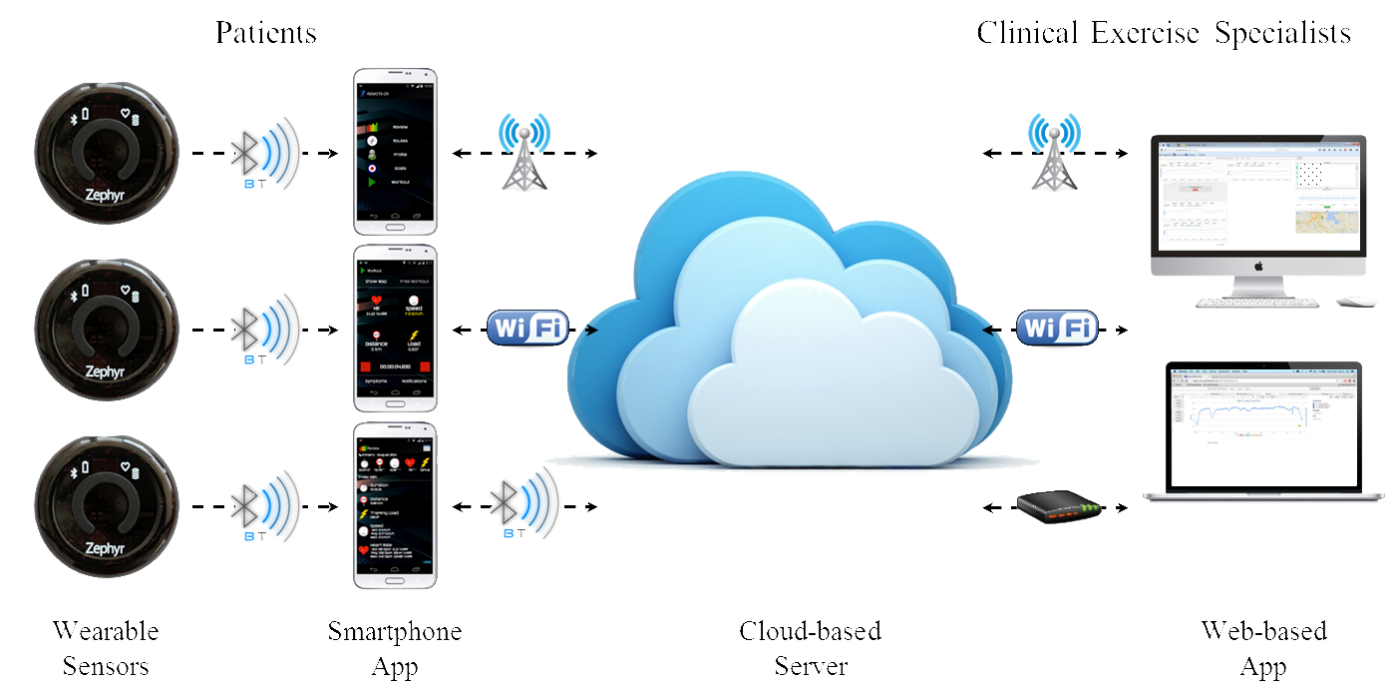
REMOTE-CR platform schematic.

The REMOTE-CR platform comprises a commercially available Android-based smartphone mobile phone and wearable sensor, custom smartphone and Web-based apps, and a custom middleware platform (Figure 1).

Smartphones were considered to be the optimal communication platform as near ubiquitous mobile broadband availability in many developed countries allows mHealth exCR to be delivered to patients in almost any location. The Android smartphone operating system (Google Inc., USA) was selected as the basis for the REMOTE-CR smartphone app as it has a majority share of the smartphone operating system market [56] and the widest range of handsets, including many low-priced models. These attributes ensure REMOTE-CR could be accessible to a broad range of patients, and will aid translation into practice.

The BioHarness 3 wearable sensor (Zephyr Technology, USA) was selected because its multisensor array is well suited for monitoring exercise among CHD patients, inbuilt Bluetooth connectivity enables integration with almost all current smartphones and commercial availability will assist accessibility and translation into practice. The BioHarness sensor array quantifies electrocardiogram (ECG, including waveform data), heart rate, heart rate variability, respiratory rate, torso posture, and triaxial acceleration. The capability to monitor heart rhythm in addition to the more ubiquitous heart rate, and respiratory rate were considered advantageous for monitoring cardiovascular workload during exercise among CHD patients. While previous versions of the BioHarness have been validated [57-62], a validation of the BioHarness 3 was conducted as part of the REMOTE-CR development process [35].

The custom software components of the REMOTE-CR platform include a middleware named Odin, a patient-facing Android smartphone app, and a clinical exCR specialist-facing Web-based app. All software components were designed and built by the research team. The Odin middleware links smartphone and server-side software to manage communication and logistic functions including data security, network connectivity, and device resource usage [63,64]. By addressing low-level functionalities, Odin enabled development of the patient- and specialist-facing apps to focus on optimizing features for mHealth exCR. Furthermore, iterative middleware refinements can be rapidly integrated into the REMOTE-CR platform without requiring substantial changes to the smartphone or Web-based apps.

The REMOTE-CR smartphone app [65] has been built for Android version 4.0 or higher, which includes most Android smartphones released since 2012. The smartphone app underwent an iterative development process, and was included in the validation of the REMOTE-CR platform in order to determine real-time data transmission reliability [35]. The Web-based app is accessible via any Internet browser, including desktop and mobile browsers, and does not require installation of specialized software. This enables true mobile capability for both patient- and specialist-facing apps, and allows the REMOTE-CR platform to operate from any location where the patient and specialist have a mobile or local Internet connection. To address the overall design objectives, the smartphone and Web-based apps were designed to include components that enable real-time remote exercise monitoring and coaching, retrospective exercise performance review, goal setting, behavior change education, and social support. Descriptions of specific components of the smartphone and Web-based apps are provided below, and include links to the underlying theory- and evidence-based constructs. Related patient- and specialist-facing components have been described together to reinforce the connected, interactive context of the platform.

### Real-Time Remote Exercise Monitoring and Coaching

Exercise monitoring and coaching capabilities have been modeled on center-based exCR, where exCR specialists provide patients with face-to-face supervision, individualized coaching, behavior change education, and social support. Real-time remote exercise monitoring and coaching allows more responsive and individualized management of patients’ exercise training in comparison to previous telehealth exCR platforms, which commonly enable only periodic interaction between specialists and patients, based on asynchronous measurement of physical activity level or exercise adherence. Responsive supervision and coaching will help maximize the benefits of exercise with gradual and appropriate progression of exercise prescription parameters, and teach patients how to manage their exercise within suitable ranges of duration and intensity level [20].

**Figure 2:**
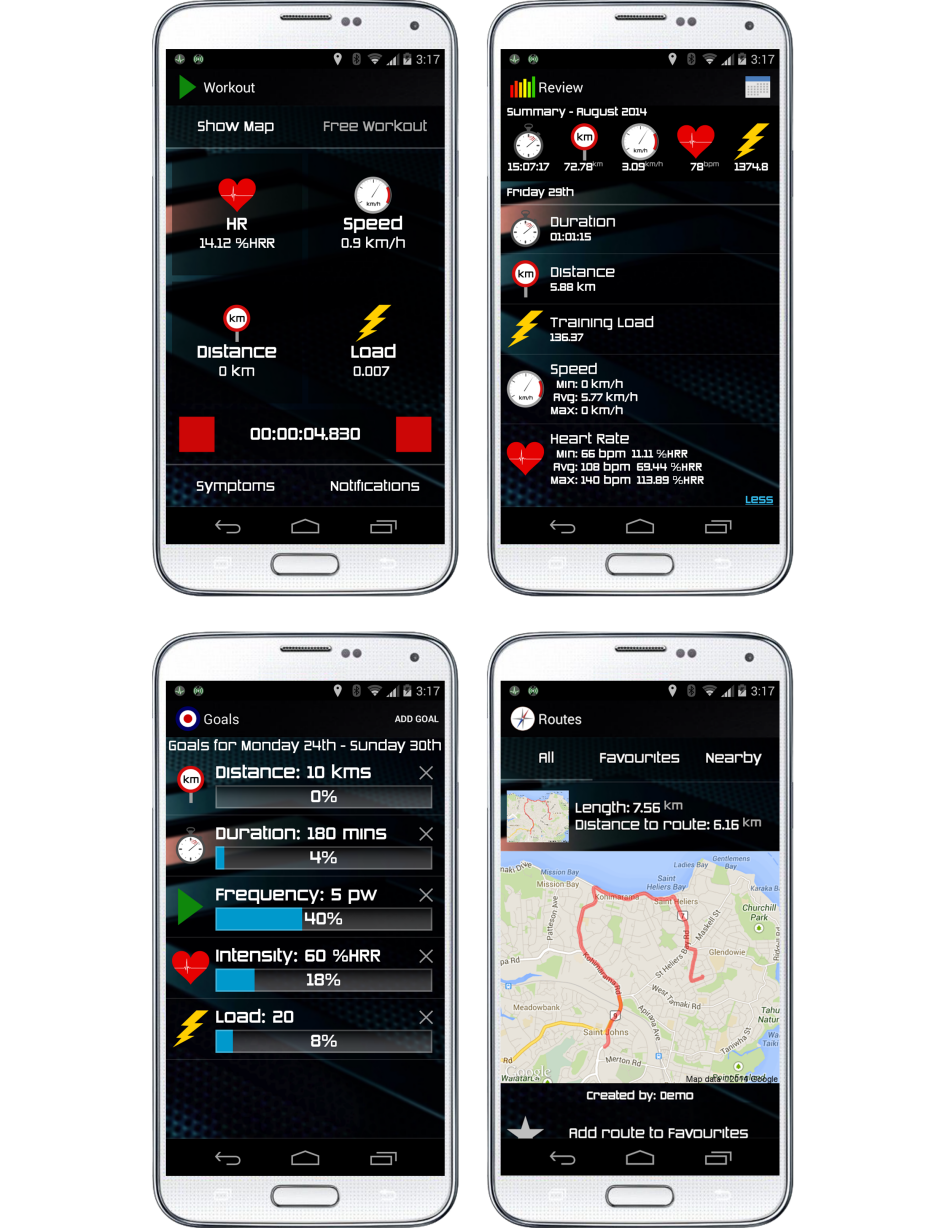
REMOTE-CR mobile phone app screenshots.

A dedicated exercise monitoring component of the patient-facing smartphone app receives wearable sensor data via Bluetooth. Sensor data are displayed continuously alongside geolocation data (provided by the smartphone) and an aggregate measure of training load (ie, exercise dose) [66]. The display configuration can be customized to suit patients’ preferences (Figure 2). Patients initiate real-time data transmission to a secure remote Web server when ready to begin exercise, and are greeted with a message from exCR specialists that includes individualized exercise prescription parameters and behavior change content. Patients continue to receive individualized messages from exCR specialists via the smartphone app throughout exercise; messages include coaching, feedback about adherence to individualized exercise prescription parameters, encouragement, and social support.

Real-time interaction allows responsive modification of exercise behavior and provision of individualized competence information that supports exercise-related confidence (ie, self-efficacy, perceived competence). Immersive real-time social support is expected to enhance perceptions of relatedness. Remotely monitored exCR inherently supports perceived autonomy in a way that face-to-face supervision may not, as patients will develop exercise behaviors in real-world environments that remain accessible beyond the program duration. This may streamline the transition to independent exercise, and promote long-term exercise adherence.

Patients can report cardiovascular symptoms including angina, dyspnea, and light headedness during exercise, using an accepted clinical symptom rating scale that has been adapted for use on smartphones [67]. ECG waveform data are not transmitted continuously in order to manage mobile broadband bandwidth requirements, but all symptom reports are accompanied by an ECG rhythm strip and exCR specialists can access on-demand ECG data whenever required via the Web-based app.

**Figure 3:**
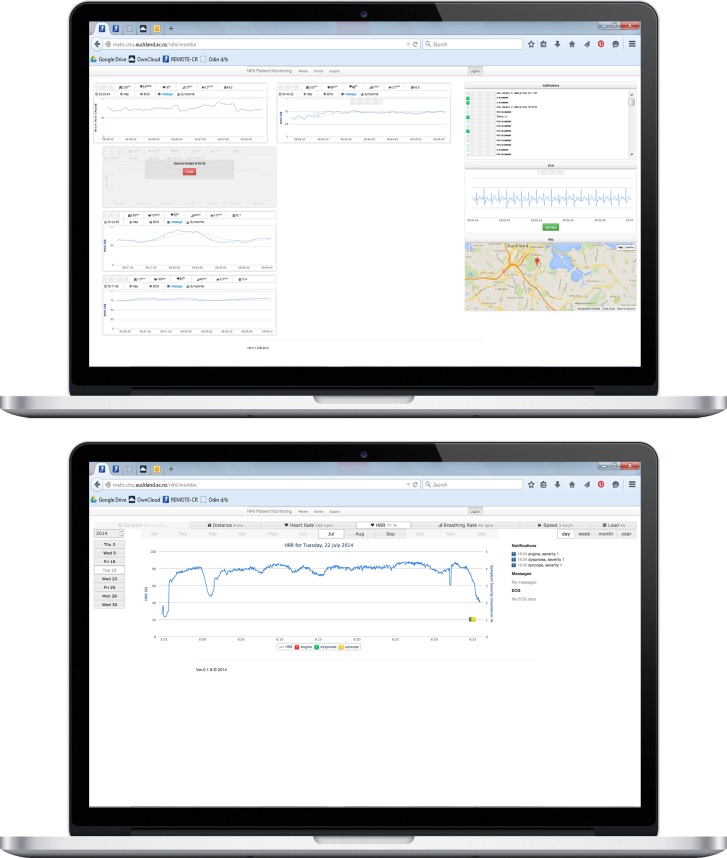
REMOTE-CR web-based app screenshots.

The exercise monitoring component of the specialist-facing Web-based app continuously retrieves patients’ exercise data from a secure Web server and visualizes them for real-time review. ExCR specialists view patient data within individual monitoring panes that display instantaneous data for all exercise variables and a configurable time-series graph to enable trend analysis. Simultaneous monitoring of multiple patients is supported. Exercise intensity level is displayed as a percentage of heart rate reserve (%HRR, the proportional difference between rest and maximal exercise heart rates) [68], which is a more accurate indicator of metabolic demands than the percentage of maximal heart rate [69,70]. Furthermore, this method simplifies individualization of exercise prescription as exCR specialists are not required to recall resting and maximal exercise data for each participant. If required, parameters that underpin %HRR can be edited in the smartphone app. Separate monitoring panes in the Web-based app display patients’ location, notification events, and on-demand ECG data (Figure 3). Location monitoring enables exCR specialists to consider effects of terrain on exercise responses, integrate local terrain into exercise prescription management, and provide accurate information if an emergency response is required. Notification events include newly initiated exercise training sessions, individualized exercise threshold alerts, and patient-reported cardiovascular symptoms. Exercise alert thresholds can be used to identify patients who are noncompliant with prescribed exercise parameters [67] and improve efficiency during multipatient monitoring by allowing exCR specialists to prioritize attention on patients who need the closest supervision.

The Web-based app enables exCR specialists to provide patients with real-time individualized coaching, feedback, social support, and instructions related to cardiovascular symptoms via a text-to-audio messaging system. Written messages are processed by a text-to-audio converter before being presented to patients in real time, typically via earphones. Audio messages will enhance patients’ perceptions of relatedness as they can closely approximate interaction provided during center-based exCR. Audio messages help preserve the real-time context of the message content, and enhance usability by eliminating the need for patients to manually check message content via the smartphone. Patients can manually replay audio messages and review message text during exercise, if required.

Real-time exercise monitoring and coaching features integrate behavior change techniques [45] including behavior goal setting, feedback on behavior, biofeedback, social support (unspecified and emotional), instruction on how to perform a behaviour, information about health consequences, behavioral practice/rehearsal, graded tasks, credible source of information, social reward, and verbal persuasion about capability.

### Evidence-Based Clinical Exercise Prescription

Exercise intervention content is informed by evidence-based guidelines for prescribing exercise to cardiac patients [20,67]. Consistent with American College of Sports Medicine guidelines [67], the REMOTE-CR exercise program comprises three training sessions per week for 12 weeks, and encouragement to be active on most week days (ie, ≥ 5 days). Exercise prescription parameters are individualized and progressed based on patients’ maximal aerobic exercise capacity (⩒O_2_max), exercise-induced signs and symptoms, age, sex, and exercise tolerance throughout the program. Patients with higher baseline ⩒O_2_max and no inducible signs or symptoms begin with a more challenging exercise prescription than those with lower capacity and/or inducible signs or symptoms. Exercise duration ranges from 30–60 min and includes warm-up and cool-down phases to allow appropriate cardiovascular and musculoskeletal preparation and recovery. Exercise intensity level ranges from 40–65% HRR, and is adjusted to optimize physiological adaptations while remaining below a metabolic load that induces abnormal clinical signs or symptoms (if present). REMOTE-CR was designed to focus on aerobic exercise modes such as walking, cycling, and stationary ergometry (eg, rowing, elliptical training). Underwater activities are not supported as wireless communications (eg, Bluetooth, mobile broadband) do not function underwater. Strength and neuromuscular training are recommended components of exCR [67] but existing sensor technologies are incapable of quantifying these modes; this remains an important challenge for mHealth exCR to overcome. The REMOTE-CR platform architecture has been designed to enable rapid integration of additional data sources whenever the appropriate sensor technologies become available.

### Exercise Performance Review

The patient-facing smartphone app and specialist-facing Web-based app both include dedicated components for retrospectively reviewing exercise performance data. Both components synchronize with a secure Web server; data presentation varies to suit differing needs of patients and exCR specialists. The patient-facing smartphone app summarizes exercise duration, distance, speed, heart rate reserve, training load, received messages, reported symptoms, and location for each training session. Information is also aggregated over calendar months (Figure 2).

The specialist-facing Web app allows exCR specialists to view full resolution data for each training session, including cardiovascular symptoms and ECG, and summarizes patients’ compliance with individualized exercise prescription parameters (Figure 3). These data allow exCR specialists to review patients’ adherence to exercise prescription goals, can inform decisions about the individualized progression of exercise prescription parameters, and help to identify patients who may need additional support. Web-based app performance review features also allow exCR specialists to review data recorded outside scheduled real-time monitoring operating hours. This provides additional flexibility for patients and may help to sustain regular exercise, while still enabling exCR specialists to track patients’ performance. Similar to previous telehealth exCR programs, specialists are able to communicate with patients who predominantly exercise outside real-time monitoring windows via telephone, SMS, or email.

Patient- and specialist-facing exercise performance review features provide information that will inform patients’ perceptions of self-efficacy and competence, and integrate behavior change techniques [45] including reviewing behavior goals, monitoring behaviours without feedback, feedback on behaviour, self-monitoring (behavior, outcomes of behavior), and biofeedback.

### Goal Setting

Goal setting components are included in both the patient-facing smartphone app and the specialist-facing Web-based app. Patients are able to set individualized weekly goals related to exercise parameters such as duration, frequency, distance, level of intensity, and training load (Figure 2). Goal achievement feedback is automated based on recorded exercise performance data; visualized feedback is updated weekly to reflect current goal achievement. Goal setting, though not compulsory, is encouraged during the initial training module and throughout the program via behavior change education content (described below). Patients can create, edit, and remove goals at any time.

ExCR specialists can set goals in conjunction with individualized exercise prescription and/or physical activity recommendations. Visual feedback is presented for short- and long-term goal achievement in order to facilitate monitoring of patients’ adherence to prescribed exercise volumes and recommended physical activity levels.

Patient- and specialist-facing goal setting features are discrete; that is, specialists do not see patients’ goals and vice versa. This allows patients to take ownership of goal setting, and individualization will help ensure goals enhance motivation. Similarly, exCR specialists can monitor patients’ performance against evidence-based exercise prescription parameters and physical activity guidelines. This helps exCR specialists to optimize exercise prescription, coaching, and behavior change content for individual patients. Patient and specialist goals can be reset or modified as required to align with progress throughout the program and maintain an optimal motivational effect.

Goal setting features support patients’ perceptions of autonomy, competence, and self-efficacy, and integrate behavior change techniques such as goal setting (behaviour, outcomes of behaviours), reviewing behavior goals, and reviewing discrepancies between current and goal behaviors.

### Behavior Change Messages

A suite of behavior change messages are delivered to patients as audio messages throughout the exercise program. Messages are received during exercise, and can be reviewed at any time as part of the exercise performance review features described above. Message content was adapted from previous mHealth CR interventions designed for delivery via SMS [71,72] that have demonstrated positive effects on lifestyle behaviors [27,73], was grounded in behavior change theories, and integrated feedback from CR patients [55]. SMS message tone was adapted for the real-time conversational context of REMOTE-CR, and also took advantage of the larger character allowance to include more natural language. Messages aimed to enhance patients’ perceptions of exercise self-efficacy, competence, and relatedness, and integrated behavior change techniques [45] including problem solving, outcome goal setting, action planning, reviewing behavior goals, self-monitoring (behavior, outcomes of behavior), social support (unspecified and emotional), instruction on how to perform a behaviour, information about health consequences, generalization of a target behavior, information from a credible source, self-reward, framing/reframing, verbal persuasion about capability, and focus on past success.

### Social Support

In the current REMOTE-CR implementation, social support is provided via real-time exercise coaching and behavior change messages, as described above. A dedicated social support component was also designed, but has not yet been implemented. Social support, from other cardiac patients in particular, is a highly valued aspect of CR [55]. Smartphone app features were designed to enable patients to interact with each other, both via the REMOTE-CR platform and face-to-face. Prototype social support features include a secure, closed social network, and location aware filtering of exercise route mapping (Figure 2). Social network functionality was designed to allow both individual and group communication in order to share experiences, provide encouragement, discuss queries or problems, offer advice, and organize face-to-face meetings if desired. Location aware filtering of exercise route maps was designed to help identify patients who exercise in close proximity to each other, and promote opportunities to exercise together and/or organize face-to-face meetings.

Social support features were designed to support patients’ perceptions of self-efficacy and relatedness, and integrate behavior change techniques [45] including social support (unspecified, emotional).

### Security

To ensure privacy, the REMOTE-CR platform requires user accounts to be registered into a database on a secure Web server; access to the database is limited to the study team. The patient-facing smartphone app is publicly available via the Google Play Store [65]. Smartphone and Web-based app functionalities are contingent on authentication with the secure Web server to prevent unauthorized use of the REMOTE-CR platform, and all data transmission is encrypted. Planned improvements include integrating the secure hypertext transfer protocol for smartphone–Web-server communication, and the OAuth protocol for Web-based app–Web server communication.

## Discussion

This article outlines the development of an evidence- and theory-based mHealth exCR platform that provides real-time remote exercise monitoring and coaching, social support, and behavior change education. Rather than redesigning the fundamental exCR process, REMOTE-CR aimed to close the current gap between center- and home-based exCR programs by mobilizing the expertize of exCR specialists. Advanced wearable sensor and smartphone technologies overcome common accessibility barriers that limit center-based exCR participation, while preserving clinical oversight that is commonly recommended in exCR guidelines [4,20,39]. In doing so REMOTE-CR provides an alternative delivery model that may help to broaden the reach of exCR by meeting the needs of patients who are unable or unwilling to attend traditional center-based exCR programs. It should be noted that REMOTE-CR was not designed to replace existing exCR programs; rather, it was designed to complement center-based programs, and may augment home-based programs that do not currently provide clinical exercise supervision.

### Principal Findings

REMOTE-CR builds on previous telehealth exCR platforms that have commonly relied on periodic telephone, email, or SMS interaction between patients, and exCR specialists and/or clinicians [21-31]. By enabling more responsive (ie, real-time) and individualized management of patients’ exercise programing, and more immersive social support during exercise, the REMOTE-CR platform may provide a more engaging environment that promotes positive motivational, behavioral, and physiological outcomes.

While real-time exercise monitoring and coaching was the primary design focus, the REMOTE-CR platform also includes a strong theoretical foundation. Many elements of the platform design and intervention content are grounded in behavior change theories that aim to enhance patients’ self-efficacy, competence, autonomy, and relatedness. This empowering approach is expected to build confidence and resilience [20], which may favor sustained positive exercise behaviors throughout and beyond the duration of the exCR program. Further, adoption of evidence-based clinical exercise prescription and monitoring guidelines [67] will help to ensure patients accrue a sufficient exercise dose to stimulate positive physiological adaptation, and provide attainable exercise targets that will support perceived self-efficacy and competence via performance accomplishments [55].

The REMOTE-CR platform was designed to enable rapid and cost-effective scalability. Rural and remote populations have reduced access to specialist services [74] and use of Internet-based services allows distribution of clinical expertise from centralized (often metropolitan) exCR facilities across geographically diverse populations. Centralized management of geographically diverse platform deployments may be a more cost-effective way to increase the reach of exCR compared with establishment of additional center-based facilities. In addition to staff time, hardware (ie, smartphone and wearable sensor) and mobile broadband subscriptions are the main operating costs. Increasingly ubiquitous smartphone ownership and mobile broadband access [32-34], and declining mobile broadband costs [75] may reduce operating costs in future; the REMOTE-CR smartphone app [65] can be installed on patients’ personal smartphones (which may promote usability), and the bandwidth requirements are low (approximately 2–4 MB·h^-1^). Use of a commercially available wearable sensor also enhances scalability.

### Limitations

While the theory- and evidence-based REMOTE-CR platform provides exciting opportunities to improve the provision and uptake of exCR services, it is not free from limitations. The REMOTE-CR platform achieved the primary objective to provide comprehensive exercise training and behavior change features, but does not currently include all core CR components recommended by international guidelines [4,39]. The platform architecture has been designed to enable modular integration of additional CR components in future, and may be expanded to create a more comprehensive CR program. At present, patients may still access additional CR components via traditional channels such as outpatient clinics and group seminars. Learning to use the smartphone and wearable sensor components of the platform represent potential barriers for CHD patients who are typically of older age. The smartphone app user interface was designed to simplify navigation, and a dedicated training module was developed to familiarize patients with the technologies; however, it is possible that some patients may not be able to overcome the technological barriers. Increasing smartphone use among older age groups [34] suggests this may be a relatively short-term barrier, but reinforces a need to retain existing exCR services to provide options for all patients. It is difficult to keep pace with rapidly advancing smartphone and wearable sensor technologies. The REMOTE-CR smartphone app was initially designed for Android 4.0, but remains compatible with all subsequent updates (v4.0–5.1). Moreover, the flexible platform architecture enables rapid integration of new smartphone and wearable sensor capabilities as they become available.

The development framework adopted for this work recommends mHealth platforms should be rigorously evaluated in studies that use randomized controlled trial design and include patient-centered outcomes to evaluate physical, mental, and social health outcomes [38]. The REMOTE-CR platform is being evaluated in a noninferiority randomized controlled trial comparing mHealth and center-based exCR programs [36]. The study will assess the effectiveness and acceptability of the REMOTE-CR platform among a CHD population; study outcomes include ⩒O_2_max, modifiable cardiovascular risk factors (blood pressure, blood lipid and glucose concentrations, body composition, physical activity energy expenditure), exercise-related motivational factors, HRQoL, and satisfaction with the REMOTE-CR platform. The results of this study will help to determine whether real-time remotely monitored exCR can provide similar benefits compared with center-based programs and, therefore, whether it represents a suitable alternative for patients whose needs are not met by existing services. If proven effective, remotely monitored exCR may offer a more cost-effective model for expanding the reach of exCR compared with the establishment of additional center-based facilities. Mobile technologies provide an ideal platform for the provision of real-time exercise-based CR and could easily be applied to other chronic disease prevention and management.

## Abbreviations

CHD: coronary heart disease
CR: cardiac rehabilitation
ECG: electrocardiogram
exCR: exercise-based cardiac rehabilitation
HRR: heart rate reserve (difference between resting and maximal heart rates)
HRQoL: health-related quality of life
mHealth: mobile health
REMOTE-CR: remote exCR platform
SMS: short message service
⩒O_2_max: maximal aerobic exercise capacity
